# Size, weight, and expectations

**DOI:** 10.1177/03010066221087404

**Published:** 2022-03-30

**Authors:** Jeroen B.J. Smeets, Kim Vos, Emma Abbink, Myrthe Plaisier

**Affiliations:** 1190 Vrije Universiteit Amsterdam, Amsterdam, The Netherlands; 1190 Vrije Universiteit Amsterdam, Amsterdam, The Netherlands; 1190 Vrije Universiteit Amsterdam, Amsterdam, The Netherlands; Delft University of Technology, Delft, The Netherlands; 200733 Eindhoven University of Technology, Eindhoven, The Netherlands

**Keywords:** vision, haptics, sensory integration

## Abstract

The size-weight illusion is well-known: if two equally heavy objects differ in size, the large one feels lighter than the small one. Most explanations for this illusion assume that because the information about the relevant attribute (weight itself) is unreliable, information about an irrelevant but correlated attribute (size) is used as well. If such reasoning is correct, one would expect that the illusion can be inverted: if size information is unreliable, weight information will be used to judge size. We explored whether such a weight-size illusion exists by asking participants to lift Styrofoam balls that were coated with glow in the dark paint. The balls (2 sizes, 3 weights) were lifted using a pulley system in complete darkness at 2 distances. Participants reported the size using free magnitude estimation. The visual size information was indeed unreliable: balls that were presented at a 20% larger distance were judged 15% smaller. Nevertheless, the judgments of size were not systematically affected by the 20% weight change (differences < 0.5%). We conclude that because the weight-size illusion does not exist, the mechanism behind the size-weight illusion is specific for judging heaviness.

## Introduction

The size-weight illusion is an illusion that has been studied scientifically for more than 130 years (Charpentier, 1891; Flournoy, 1894; Koseleff, 1937). In this illusion, people perceive the smaller of two equally weighted objects to be heavier. The illusion is extremely robust: it occurs when the size information is provided haptically or multisensory (Ellis & Lederman, 1993), but also when the size information is only available visually (Buckingham & Goodale, 2010), and also occurs when objects are lifted via strings and pulleys (Kawai et al., 2007; Pant et al., 2021; Pick & Pick, 1967; Vicovaro & Burigana, 2017). The effect of visual size on weight judgements does not reflect the estimated amount of material in an object (Plaisier & Smeets, 2015) but reflects a judgement of its overall size. A size-weight illusion is therefore also present when objects have the same size but differ in apparent size due to a visual illusion (de Brouwer et al., 2016). Although the attribute ‘weight’ refers to gravity, the illusion works independent of gravitational forces (Plaisier & Smeets, 2012), so it is actually a size-mass illusion.

The fact that the illusion can be (un)learned after prolonged training (Flanagan et al., 2008) indicates that the illusion is based on a strong association between size and weight in everyday situations. This is perfectly in line with the most common explanation of the illusion: weight is judged relative to expected weight. This interpretation is also in line with the related material-weight illusion: an object that appears to be made of material with a higher density feels lighter (Ross, 1969), and with the report that the size-weight illusion is sensitive for social properties (Dijker, 2008). However, our recent finding that visual size can affect judged weight even if it becomes available after lifting has started (Plaisier et al., 2019) is in conflict with this theory. Several other explanations have been proposed (Buckingham, 2014; Dijker, 2014; Wolf et al., 2018).

The size-weight illusion is remarkably strong: in earlier studies we have found that for a 1-cm increase in size of a 5-cm long object (20%), the perceived mass decreased from 180 by 12 grams (Plaisier & Smeets, 2015). For a 30% increase in length, we reported even a 20% decrease (Plaisier & Smeets, 2012). The effect of size on mass can thus have an effect that is more than 50% of a corresponding mass change. Given the multisensory nature of the illusion, one could argue that a Bayesian or anti-Bayesian (Brayanov & Smith, 2010; Peters et al., 2016) integration of sensory information underlies the illusion. The direct sensory information about heaviness (force) is unreliable (Weber fraction >>0.1 for forces of a few Newton; Vicentini et al., 2010). This lack of reliability makes it useful to combine this direct information with other information that are expected to affect force (Diedrichsen et al., 2007). Following this reasoning, one would expect that similar illusions would occur for other attributes for which the direct information is unreliable. According to the Bayesian integration theory, one can only find the symmetric effect if the reliability of both information sources is comparable. For instance, Alais and Burr (2004) could find the inverse of the ventriloquist effect once they reduced the reliability of the visual information considerably. In this paper, we address the question whether we can find the inverse of the size-weight illusion (the perceived size of an object decreases with its heaviness) in a situation in which visual size information is made unreliable by asking participants to judge the size of isolated objects in the dark.

The question is thus whether the inverse of the size-weight illusion exists: a weight-size illusion? Several authors have tried to answer the question whether there is a weight-size illusion and obtained conflicting results. In the first study we know of, the experimenter placed two spheres on the palms of the participant's hands and asked the participant to report which of the two spheres had a larger volume, based on passive tactile information only (Usnadze, 1931). He found that in the majority of the cases, the heavier object was judged smaller. In two other studies, again without vision, but now with active movement of the participants, the opposite effect was found (Bergman, 1970; Hirsiger et al., 2012). The reason for this positive correlation is that the torques and forces experienced during movements while holding an object are perfectly suited to estimate the size of an object (Debats et al., 2010; Kingma et al., 2004; Solomon et al., 1989).

We therefore decided to answer the question using a different approach. Analogous to the classic size-weight illusion, we started each trial with providing the indirect information (heaviness) by letting the participants lift the object through a pully system. Once the object was lifted it became visible, but the size information was unreliable because we performed the experiment using glow-in-the dark objects in a completely dark room. In line with our experiments in the size-weight illusion (Plaisier & Smeets, 2012; Plaisier & Smeets, 2015), we varied both the size and the weight in the experiment, so that we could express the effect size in physical rather than statistical terms.

## Method

### Participants

Fourteen volunteers (2 male, 12 female), aged 26 ± 10 (mean ± standard deviation) years participated in the study. Five of them were bachelor students and received course credit in exchange for their participation, the others were real volunteers. All participants were right-handed and used this hand in the study. They had no known neurological deficits, and normal or corrected-to-normal vision. As we do not have an expectation about the strength of a potential weight-size illusion, we cannot provide a strong justification of the number of participants. We therefore chose the number of participants to be slightly higher than we used on our studies on the size-weight illusion using free magnitude estimation (Plaisier et al., 2019; Plaisier & Smeets, 2012; Plaisier & Smeets, 2015). To increase the power further, the participants of our present study performed twice the number of trials per condition compared to those in our previous studies.

### Set-up and Procedure

We designed the experiment in analogy to the design of earlier experiments on the size-weight illusion (Plaisier & Smeets, 2015). We used four polystyrene balls with an eye bolt to which the experimenter could attach a hook that was attached to a thread. Each ball also contained a vertical cylindrical hole that was filled to obtain the correct weight. We used three of them (10 cm diameter, weight 160, 200 or 240 gram) to test whether the mass of the ball affected the perception of size. We added a fourth ball that differed in size from the other three (12 cm diameter, 200 gram) as a reference to be able to assign a value in centimetres to a possible effect of the mass on perceived size. The balls were covered with papier mâché and painted with glow-in-the-dark paint.

**Figure 1.**
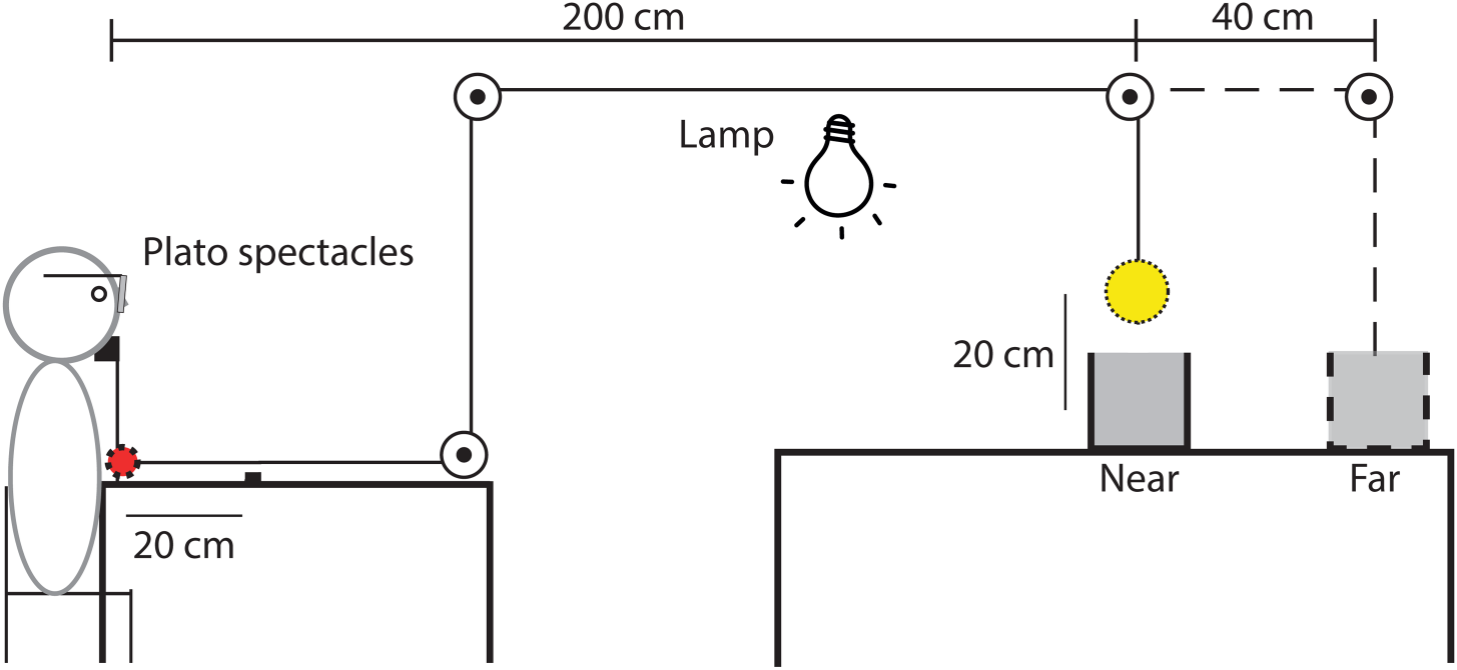
The experimental set-up. A ball (yellow) was connected to a handle (red) via a thread and pulleys. The ball was resting in either in the Near box (continuous line as drawn) or Far box (dashed line). The task of the participant was to drag the handle over 20 cm towards her (dotted line) to lift the ball above the box and report the size of the ball. The Plato spectacles open only when the Lamp is switched off.

To minimise depth cues (Brenner & Smeets, 2018), participants lifted the glow-in-the-dark balls in complete darkness. They lifted the balls using a thread via a pulley system (Figure 1) to ensure that participants could not get haptic information about object size or depth. The pulley system added frictional forces; we measured the forces used to lift the balls outside the experiment, and obtained 2.0, 2.6 and 3.0 N (standard deviation 0.2N). To ensure that the weight information was predictive (i.e. available before any visual size information), we placed the balls in a box so that they were invisible to the participants. The inside of the box was covered with cotton wool to prevent sounds that might serve as cues to identify the balls. We provided a chinrest to ensure that the participants kept their head at the desired position. To check to what extent participants were able to use depth cues, we presented the balls at two distances from the participant: 200 and 240 cm. The difference in distance is 20%, so the retinal image size of the large ball at the far distance equals that of the small ball at the short distance. To prevent seeing the experimenter manipulate the balls, participants wore Plato spectacles (Translucent Technologies Inc., Ca). They were only open at the time that participants were asked to lift the ball.

The experiment was performed in a single session and controlled by two experimenters. The participant put on the Plato spectacles before entering the room and did thus not see the set-up. One experimenter guided the participant to a chair; at that moment the room was lit to facilitate the experimenter. After the participant was seated, the experimenter handed her a handle that was connected to the thread and guided her hand to a tactile marker on the table. The experimenter explained the procedure and the task. Balls were presented individually, and participants judged their size using free magnitude estimation (Zwislocki & Goodman, 1980). For participants who had problems with the freedom to choose a reference, the experimenter explained that the participant was free to judge the size in centimetres. Each participant performed eight practice trials in which they experienced each combination of ball and position once. In this way they got accustomed to the task and could establish a scale for the free magnitude estimation.

Each trial started with one experimenter connecting one ball to the thread at one of the two distances, while the participant held the handle at a tactile marker on the table. The other experimenter turned off the light, opened the Plato spectacles and a beep sounded. The participant lifted the ball 20 cm by moving her hand towards their body until they touched a second tactile marker so that the ball was completely visible, judged the ball's size, and moved her hand back to the far marker. The participant had three seconds to perform this procedure. At that time, the Plato spectacles were closed, and the room light was turned on. In the closed state, the Plato spectacles are milky (they transmit light without providing any information), which prevented dark adaptation of the participants. This ensured that participants could only see the ball when the spectacles opened. Each combination of ball and position was repeated 21 times, resulting in a total of 168 trials that were presented in random order. The total duration of the experiment was about 50 min, including a break of about 2 min halfway the experiment. During the break, the light was turned on; the participant continued wearing the opaque Plato spectacles to prevent seeing the set-up.

### Data Analysis

To facilitate the interpretation of free-magnitude judgements, we followed the procedure we used in earlier studies (Plaisier & Smeets, 2012; Plaisier & Smeets, 2015). We subtracted for each participant the overall mean of all scores, and divided the resulting numbers by the standard deviation, resulting in z-scores. Using the difference in the ratings for the 10 and 12 cm sized objects, the z-scores were converted into centimetres. To determine whether weight had affected the size judgements, we performed a linear regression on the size judgements for the 10 cm balls with distance and weight as independent variables. A significant slope for weight would imply the existence of a weight-size illusion. A significant slope of distance would imply that participants cannot correctly scale the retinal image of the balls for the viewing distance, an effect that we deliberately promote by performing the experiment in the dark.

## Results

Our participants had no problem in reporting the size of the balls. During the debriefing of the experiment, we asked participants whether they could report the number of balls that we had presented to check whether they might have used the balls identities to consistently report size. None of the participants could. They reported that it was difficult to recognize the balls because they were generally rotating when they became visible. The precision of the size judgements of individual participants (the SEM of the 21 judgements) was on average 0.27 cm for the 12 cm balls and 0.24 cm for the 10 cm balls. The average precision of an individual participant ranged from 0.14 to 0.43 cm.

**Figure 2.**
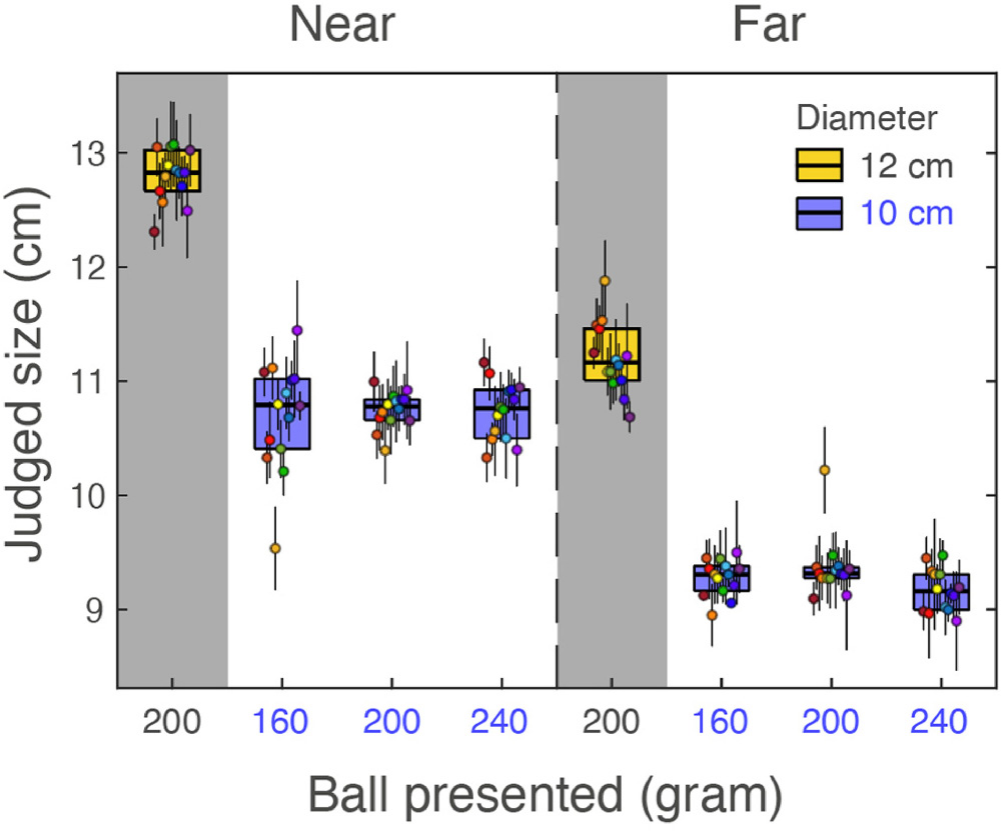
The judged size for the four different balls at two distances (left and right half of the plot). We plotted the main results (for the three 10 cm diameter balls that differed in weight) on a white background, and those for the 12 cm diameter reference ball on a grey background. The colored disks indicate the average settings of the various participants (color coded) with standard error. The rectangles indicate the inter-quartile range around the median (thick horizontal line).

Two aspects of the results are directly visible from the data in Figure 2. The first one is that the participants reliably reported the 12 cm ball as larger than the 10 cm balls (all individual data in the grey areas are clearly above the ones in the white areas to the right). The second clear aspect is that, as expected, the participants judged the far balls as smaller than the near balls. The 10 cm balls at 200 cm distance were judged on average 0.5 cm smaller (95% confidence interval 0.2–0.7 cm) than the 12 cm ball at 240 cm. As these two situations produced the same retinal image, the size judgements were not only based on the retinal image size; we will come back to this in the discussion. These results indicate that we succeeded in our aim to reduce the visual information to an extent that participants made large errors in size judgements. Although their judgements were not reliable, they were still reproducible. Most importantly, the fact that the judgements of the size of the three 10 cm balls does not differ much (data on the white background in Figure 2) indicates that there was no large effect of heaviness on size judgements. If a weight-size illusion would exist that is comparably strong as the size-weight illusion (Plaisier & Smeets, 2015), one would expect an effect of about 1 cm.

To answer the question whether weight affects size judgements, we analysed the responses to the three balls that differed in weight using a multiple linear regression on the size judgements with distance and weight as independent variable. As expected, the slope for the dependence of judged size on distance differed from zero (−35 ± 1.9 mm/m; t = −18.4; *p* < 0.001). Most importantly, the judged size was independent of weight (slope 0.84 ± 1.17 mm/kg; t = 0.717; *p* = 0.475). The lack of a significant illusion is in line with our visual judgement based on Figure 2. Of course, a lack of a significant illusion does not imply that a very small illusory effect cannot exist, which would be orders of magnitude smaller than the size-weight illusion. However, given the positive value we obtained for the slope, such potential illusion would be most likely a very small assimilation, rather than a contrast illusion such as the size-weight illusion.

## Discussion

We questioned whether the influence of size on perceived heaviness (the size-weight illusion) is the consequence of a more general mechanism of sensory integration. If so, one would expect the reverse illusion to exist as well: the weight-size illusion. We tested whether in a situation in which judgements of size cannot be performed reliably, a prediction of its size based on the experienced heaviness would influence the size judgements. We found a very clear answer: there was no substantial weight-size illusion in our experiment. Does the fact that we did not find a weight-size illusion in our experiment imply that this illusion does not exist? There are a few potential issues that might prohibit drawing this strong conclusion.

A first potential issue is the reliability of the heaviness information. We let subjects lift the balls using a pulley system to exclude haptic information about object distance and size. This pulley system added additional (frictional) forces. We measured these forces, and on average they scale with object weight, so the effective force difference between the objects is larger with friction (1.0 N) than if they would be lifted directly (0.8 N). However, the friction adds variability to the forces that is not one-to-one related to the acceleration. Nevertheless, such pulley systems have been extensively used in studies on the size weight illusion (Anderson, 1970; Buckingham & Goodale, 2010; Ellis & Lederman, 1993; Masin & Crestoni, 1988), which all reproduced heaviness judgements that correlated well with the objects’ mass.

A second issue might be the range of masses that we employed. One could argue that if we would have employed larger mass differences, the difference in perceived size might have been bigger. We do not think that our various experiments on the size-weight illusion support this idea. In our study with the largest manipulation of size (33%), we found effects on perceived weight of about 20% (Plaisier & Smeets, 2012), so a fraction of 0.6. The experiment that involved the smallest manipulation in size was one in which we manipulated perceived size by the Müler-Lyer illusion (de Brouwer et al., 2016). This illusion made a bar appear 4% smaller, and at the same time 7% heavier. So, the smallest manipulation of size resulted in the largest (relative) effect of the illusion.

A third potential issue is the limited number of participants, and potential errors in the experiment. Looking at the individual data in Figure 2, there is one participant that reports a much smaller size for the 160 gram, 10 cm ball that was presented near, and a much larger size for the 200 gram, 10 cm ball that was presented far. Such outlier values could artificially increase the variability, and thus mask the presence of a potential illusion effect. If we would exclude this participant, the statistical conclusion remains the same: no evidence at all for the existence of a weight-size illusion.

A last potential issue is that we covered the balls with papier mâché, so that we could paint them. This means that with some effort, participants might have been able to visually distinguish between the three 10-cm balls. However, our debriefing (see results) did not provide any indication for recognition. Moreover, the fact that the judgements of the size of the balls differed so strongly between the two presentation distances makes it even more unlikely that recognition has interfered with our experiment. As none of the four issues seriously interferes with our results, we can safely conclude that no substantial weight-size illusion exists.

Our finding that our participants judged the balls smaller in the Far than in the Near location (data on the right in Figure 2 lower than those on the left) shows that they do not take the egocentric distance well enough into account. The 0.5 cm difference in the perceived size between the 12 cm ball in the far position and the 10 cm ball in the near position indicates that the distance information is only for 25% considered. As we eliminated cues such as occlusion, relative size and perspective, we think that the small difference in size is mainly due to binocular cues such as vergence and relative disparity. In addition, even with the reduced head motion by a chinrest, motion parallax might play a role in the distance judgement (de la Malla et al., 2016), just as the difference in luminance of the retinal image.

In contrast to the very strong visual size-weight illusion (vision influencing a haptic judgement), there is no considerable weight-size illusion (haptics influencing a visual judgement). This conclusion seems in line with the widespread notion of visual dominance in perceiving object properties (Rock & Victor, 1964). However, various experiments have shown that this assumed visual dominance only occurs if visual information is more reliable (Ernst & Banks, 2002; van Beers et al., 2011). We therefore made vision deliberately unreliable in the present experiment. Our results are in line with the results of another experiment investigating visual size perception in the dark. In this experiment, participants had to judge the size of a cube that was attached to a rod they were holding by comparing it with the haptic size of an invisible hand-held cube. In this situation, the kinesthetic information on the object's distance did not improve visual judgments of its size (Brenner et al., 1997).

The lack of symmetry between judgements of size and weight implies that the size-weight illusion is not a general property of expectations based on correlations between attributes. This is in line with our previous finding that the size-weight illusion also occurs when the visual information is only provided after the object is already accelerating upwards (Plaisier et al., 2019). The lack of symmetry implies furthermore that the size-weight illusion is not a general property of multisensory integration (Brayanov & Smith, 2010; Peters et al., 2016). It is in line with explanations based on the assumptions that the size-weight illusion is not related to size, but to density (Wolf et al., 2018). If the illusion is actually a density-weight illusion, irrespective of size, it means that there is no integration of size and weight information. This reasoning makes it less surprising that size is not affected by weight. Unfortunately, not all experimental results point in this direction: if objects’ sizes are manipulated in a way so that their density remains the same, the size-weight illusion is present and equally strong as usual (Plaisier & Smeets, 2015). So, our finding that there is no considerable weight-size illusion did not reduce the enigma of the size-weight illusion.
